# Deregulated expression of circadian clock genes in gastric cancer

**DOI:** 10.1186/1471-230X-14-67

**Published:** 2014-04-06

**Authors:** Ming-Luen Hu, Kun-Tu Yeh, Pai-Mei Lin, Cheng-Ming Hsu, Hui-Hua Hsiao, Yi-Chang Liu, Hugo You-Hsien Lin, Sheng-Fung Lin, Ming-Yu Yang

**Affiliations:** 1 Division of Hepatogastroenterology, Department of Internal Medicine, Kaohsiung Chang Gung Memorial Hospital and Chang Gung University College of Medicine, 123 Da-Pei Road, Niaosung District, 833 Kaohsiung, Taiwan; 2 Graduate Institute of Clinical Medical Sciences, College of Medicine, Chang Gung University, 259 Wen-Hwa 1st Road,Kwei-Shan 333 Tao-Yuan, Taiwan; 3 Department of Pathology, Changhua Christian Hospital, 135 Nan-Hsiao St., 500 Changhua, Taiwan; 4 Department of Nursing, I-Shou University, No.1, Sec. 1, Syuecheng Road, Dashu District, 840 Kaohsiung City, Taiwan; 5 Department of Otolaryngology, Kaohsiung Chang Gung Memorial Hospital and Chang Gung University College of Medicine, 123 Da-Pei Road, Niaosung District, 833 Kaohsiung City, Taiwan; 6 Division of Hematology-Oncology, Department of Internal Medicine, Kaohsiung Medical University Hospital, 100 Tzyou 1st Road, 807 Kaohsiung City, Taiwan; 7 Faculty of Medicine, Kaohsiung Medical University, 100 Tzyou 1st Road, 807 Kaohsiung City, Taiwan; 8 Division of Nephrology, Department of Internal Medicine, Kaohsiung Medical University Hospital, 100 Tzyou 1st Road, 807 Kaohsiung, Taiwan; 9 Department of Internal Medicine, Kaohsiung Municipal Ta-Tung Hospital, Kaohsiung Medical University, 68 Jhonghua 3rd Road, 801 Kaohsiung, Taiwan

**Keywords:** Gastric cancer, Circadian clock genes, Circadian rhythm

## Abstract

**Background:**

Gastric cancer (GC), an aggressive malignant tumor of the alimentary tract, is a leading cause of cancer-related death. Circadian rhythm exhibits a 24-hour variation in physiological processes and behavior, such as hormone levels, metabolism, gene expression, sleep and wakefulness, and appetite. Disruption of circadian rhythm has been associated with various cancers, including chronic myeloid leukemia, head and neck squamous cell carcinoma, hepatocellular carcinoma, endometrial carcinoma, and breast cancer. However, the expression of circadian clock genes in GC remains unexplored.

**Methods:**

In this study, the expression profiles of eight circadian clock genes (*PER1*, *PER2*, *PER3*, *CRY1*, *CRY2, CKIϵ, CLOCK,* and *BMAL1*) of cancerous and noncancerous tissues from 29 GC patients were investigated using real-time quantitative reverse-transcriptase polymerase chain reaction and validated through immunohistochemical analysis.

**Results:**

We found that *PER2* was significantly up-regulated in cancer tissues (*p* < 0.005). Up-regulated *CRY1* expression was significantly correlated with more advanced stages (stage III and IV) (*p* < 0.05).

**Conclusions:**

Our results suggest deregulated expressions of circadian clock genes exist in GC and circadian rhythm disturbance may be associated with the development of GC.

## Background

Gastric cancer (GC) is one of the leading causes of cancer-related death worldwide [1,2]. Early detection of GC often offers a better prognosis but most patients are diagnosed with GCs at late stages. Early GC, that is, cancer only invading the mucosa or submucosa without lymph node or distant metastasis, has a >90% 5 year survival rate regardless of endoscopic or surgical resection. However, the prognosis of patients with advanced GC is dismal. Delayed diagnosis at an advanced stage is often attributable to late onset of clinical symptoms, which limits available therapeutic approaches in more than 50% of cases [2-4]. Upper gastrointestinal endoscopy is the gold standard for the diagnosis of GC. Endoscopic ultrasound and abdominal computed tomography are important tools for preoperative staging. However, preoperative staging is sometimes misjudged and correct cancer staging is confirmed after surgery. Until now, no useful biomarkers have been available for the prediction of cancer stage, prognosis or treatment outcome.

The 24-hour rhythmic changes in human physiological processes and behavior are controlled by autonomous biological pacemakers, which are called circadian clocks. The regulation of circadian oscillators occurs through transcriptional-translational feedback loops, which consist of at least nine core circadian clock genes including *PER1*, *PER2*, *PER3*, *CLOCK*, *CRY1*, *CRY2*, *BMAL1*, *CK1ϵ*, and *TIM*[5-8]. Disruption of circadian rhythms is associated with cancer development and tumor progression [9-11]. Epidemiologic studies of nightshift workers have revealed that circadian disruption is a critical factor in the tumorigenesis of breast cancer [12], skin cancer [13], colorectal cancer (CRC) [14], prostate cancer [15], and endometrial cancer [16]. Innominato et al. [17] have also found that interventions to normalize circadian timing system dysfunction affect the quality of life and survival of patients with metastatic colon cancer.

Many recent studies have also demonstrated that the expression of circadian clock genes is disturbed in cancers such as hepatocellular carcinoma (HCC) [18], chronic myeloid leukemia (CML) [19,20], and head and neck squamous cell carcinoma (HNSCC) [21]. However, confirmation of an association between circadian clock genes and GC is still lacking. Therefore, in this study we studied the expression of circadian clock genes in GC aiming to find links between altered circadian rhythm and GC, and assess the usefulness of these genes as biomarkers to predict disease severity and treatment outcome.

## Methods

### Patients and samples

Cancer tissue and the adjacent noncancerous tissues were obtained from 29 patients (20 men and 9 women) aged 51–81 years (mean ± standard deviation, 69.76 ± 9.10 years) with gastric adenocarcinoma being treated with surgery at Changhua Christian Hospital (Changhua, Taiwan) between 2000 and 2002. Adjacent noncancerous tissues were obtained 1 cm apart from tumor tissue and confirmed histologically by a pathologist. Clinical characteristics including patient’s age, sex, tumor staging, and survival are listed in Table 1. Tumor staging was used according to the 7^th^ edition of TNM classification (tumor, lymph node and metastasis) of American Joint Committee on Cancer which divides GC into stage I, II, III and IV from early to advanced diseases. The GC tissue specimens were obtained at the following time points: 21 were obtained between 1000 and 1200 hours, and 8 were obtained between 1200 and 1400 hours. The specimens were obtained immediately after resection and frozen in liquid nitrogen until use. Informed consent was obtained from all patients after tissue acquisition. This study was carried out after the approval of the Institutional Review Board of Changhua Christian Hospital.

**Table 1:** Clinical characteristic of the 29 gastric cancer patients in this study

**Characteristic**	**Gastric cancer**
	**(n = 29)**
Sex	
Male	20
Female	9
Median age in years (range)	71 (51–81)
Staging	
I	3
II	3
III	15
IV	8
Survival	
> 5 years	8
< 5 years	17
Lost to follow-up	4

### Real-time quantitative reverse transcriptase-polymerase chain reaction (qRT-PCR) analysis of circadian clock genes

We selected eight circadian clock genes, including *PER1*, *PER2*, *PER3*, *CRY1*, *CRY2, CKIϵ, CLOCK,* and *BMAL1*, to study the expressions in GC. Total RNA was extracted from cancerous tissue and noncancerous tissue using TRIzol reagent (Invitrogen, Carlsbad, CA, USA) and complimentary DNA was generated with a High Capacity cDNA Reverse Transcription Kit (Applied Biosystems, Foster City, CA, USA) according to the manufacturer’s protocols. The designs of the specific forward and reverse primers and MGB TaqMan^®^ probes and the reaction conditions for the qRT-PCR of the eight circadian clock genes were carried out as previously described [19-21]. Expression of human *GAPDH* (glyceraldehyde-3-phosphate dehydrogenase) gene was used for normalizing circadian clock genes expression in qRT-PCR with an ABI 7700 Sequence Detector (Applied Biosystems). The expression levels of the circadian clock genes were normalized to the internal control *GAPDH* to obtain the relative threshold cycle (Δ*C*_T_), and the relative expression between cancerous and noncancerous tissues was calculated using the comparative *C*_T_ (ΔΔ*C*_T_) method (ΔΔ*C*_T_ = Δ*C*_T_ of cancerous tissue- Δ*C*_T_ of noncancerous tissue) or 2^-ΔΔ*C*^_T_.

### Immunohistochemical (IHC) staining

IHC staining was performed on cancerous tissues and the adjacent noncancerous tissues of GC patients. Monoclonal or polyclonal antibodies against circadian clock genes (Abcam Inc. Cambridge, MA, USA) were used as the primary antibodies. The tissue sections were incubated with primary antibodies (1:200 dilutions) for 1 hour and then incubated with biotinylated goat anti-rabbit antibodies for 30 minutes. The specific binding of the secondary antibodies to the primary antibodies was visualized using a horseradish peroxidase- diaminobenzidine staining kit (Abcam Inc.). After staining, the sections were mounted, cleared, cover-slipped, and examined using a Zeiss microscope (Zeiss, Gottingen, Germany).

### Statistical analysis

Differences in expression between two groups for each circadian clock gene were detected using a pair *t-*test, and the values of Δ*C*_T_ were used for all the statistical analyses. A Cox proportional hazard regression model was used for the analysis of circadian clock gene expression and prognosis. Two-sided *p* value was calculated and a difference was considered statistically significant if *p* value was < 0.05. All computations were performed using SPSS for Windows Release 13.0 software (SPSS, Chicago, IL, USA).

## Results

### Analysis of circadian clock gene expression in GC with qRT-PCR

**Figure 1:**
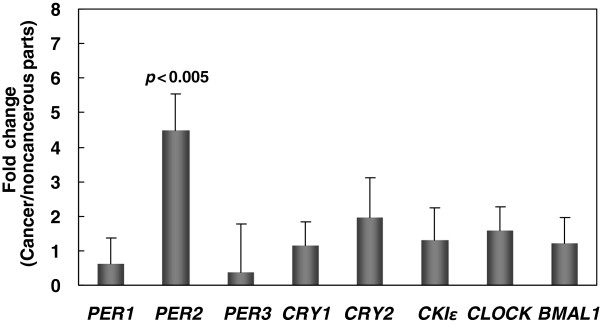
**Expression of circadian clock genes in gastric cancer (GC) determined by real-time quantitative RT-PCR.** Expression of the eight circadian clock genes in paired cancerous and noncancerous tissues from 29 GC patients. The *y*-axis represents the relative messenger RNA (mRNA) expression level. The value of mRNA expression in noncancerous tissue is designated 1, and the level of mRNA expression in cancerous tissues is calibrated to obtain the fold change in cancerous tissues. Statistical significance (p < 0.005) was evaluated with a *t*-test.

Cancerous and noncancerous tissues from 29 patients with GC were examined for the expressions of the eight circadian clock genes using qRT-PCR to elucidate whether the expression levels of circadian clock genes were deregulated in cancer tissues. Our data demonstrated that only *PER2* expression was significantly upregulated (*p* < 0.005); differences in expression between cancerous and noncancerous tissues were not statistically significant for the other seven genes (Figure 1).

### Confirmation of circadian clock gene protein expression using IHC staining

**Figure 2:**
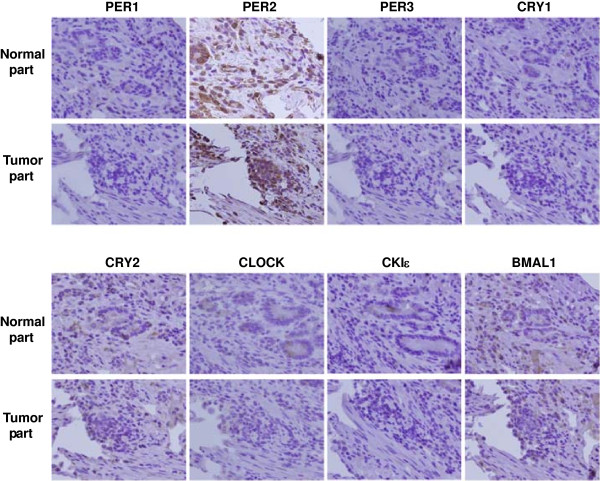
**Immunohistochemical analyses of eight circadian clock genes in gastric cancer (GC).** A representative case of GC shows higher expression of PER2 in cancerous tissues compared with that in adjacent noncancerous tissues. Expression of PER1, PER3, CRY1, CRY2, CLOCK, CKIϵ, and BMAL1 does not differ between cancerous and noncancerous tissues from GC patients. Original magnification: 400 × .

We further investigated the protein expression of the eight circadian clock genes in GC using IHC staining. IHC analysis also revealed a higher expression of PER2 proteins in cancerous tissues compared to that in adjacent noncancerous tissues. An example is shown in Figure 2 and is similar to the results of the other patients. These results confirmed the qRT-PCR observation that the expression of *PER2* was upregulated in GC tissues. The protein expression of the other seven circadian clock genes was not consistently different between cancerous and noncancerous tissues. Some circadian clock proteins could not be well detected with IHC staining in both cancerous and noncancerous tissues in some patients.

### Disease severity and circadian clock gene expression in GC patients

**Figure 3:**
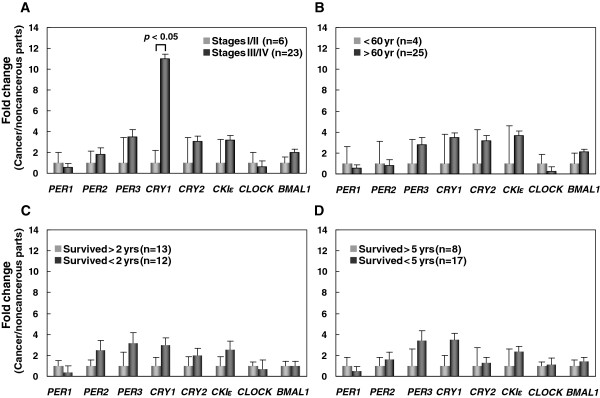
**Disease severity, age, survival and circadian clock gene expression in gastric cancer (GC) patients.** Disease severity **(A)**, age **(B)**, and survival **(C, D)** of 29 GC patients were correlated to the expression of eight circadian clock genes. The *y*-axis represents the relative messenger RNA expression level. The relative expression in cancerous tissues is calculated by ΔΔ*C*_T_. The expression in stage I/II **(A)**, age < 60 years **(B)** survival > 2 years **(C)**, and survival > 5 years **(D)** is designated 1 and the relative expression in stage III/IV **(A)**, age > 60 years **(B)**, and survival < 2 years **(C)**, and survival < 5 years **(D)** is calibrated to obtain the fold change, respectively. Statistical significance (*p* < 0.05) was evaluated with a *t*-test.

We divided the patients into earlier stages (stages I and II) and more advanced stages (stages III and IV) for correlation analysis with circadian clock gene expression and found that *CRY1* expression was upregulated in more advanced cancer stages (*p* < 0.05) (Figure 3A). The expression of the other seven circadian clock genes was not correlated with GC disease severity.

### Age and circadian clock gene expression in GC patients

To rule out the possibility that the altered circadian clock gene expression was due to age differences, we divided the patients into two groups (< 60 years-old and > 60 years-old) for correlation analysis with circadian clock gene expression. The expression of the eight circadian clock genes was not significantly different between the two groups (Figure 3B).

### Survival and circadian clock gene expression in GC patients

The survival status of the patients was followed up for 5 years after surgery. Among the 29 patients, 12 died from the disease within 2 years, 17 died within 5 years, 8 survived longer than 5 years, and 4 lost to follow up (Table 1). The correlation between the 2-year or 5-year survival status and circadian clock gene expression was further analyzed. None of the eight circadian clock genes was correlated with either the 2-year or the 5-year survival (Figure 3C and 3D). However, Cox proportional hazard regression model analysis has revealed that patients’ survival days are correlated with the expression level of *PER3* (Odd ratio = 0.901, 95% confidence interval: 0.815-0.997, *p* = 0.044 ) but not with the other seven circadian clock genes (Table 2).

**Table 2:** Cox proportional hazard regression model analysis for survival days and circadian clock gene expression in the 29 gastric cancer patients

**Relative expression of circadian clock gene**	**Hazard ratio**	**95% CI**	***p *** **value**
*PER1*	1.183	0.971-1.441	0.096
*PER2*	0.945	0.798-1.120	0.516
*PER3*	0.901	0.815-0.997	**0.044**
*CRY1*	0.879	0.761-1.015	0.079
*CRY2*	0.926	0.809-1.061	0.268
*CKIϵ*	1.024	0.873-1.252	0.819
*CLOCK*	0.891	0.706-1.124	0.328
*BMAL1*	0.917	0.802-1.049	0.206

The relative expression of circadian clock genes in cancerous tissue/noncancerous tissues was calculated by 2^-ΔΔ*C*^_T_. CI: confidence interval.

## Discussion

GC is a major health issue and remains a leading cause of cancer death worldwide. Although early GC has a good prognosis, most patients are diagnosed at advanced stages with dismal outcome. To improve the survival in GC patients, early detection and subsequent surveillance are essential. Endoscopy with biopsy is the gold standard in currently available screening and diagnostic tools. Future studies should focus on the incorporation of molecular biomarkers into clinical management to forecast the cancer stage, prognosis and improve outcome, especially in advanced-stage GC patients.

Circadian rhythms are endogenously generated rhythms that occur with a periodicity of approximately 24 hours and play an important role in regulating the daily rhythms of human physiology and behaviors. The disruption of circadian rhythms is considered a contributory factor in many clinical conditions including sleeping disorders, gastrointestinal diseases, metabolic syndrome, inflammation and even cancers [22]. Observational studies have revealed that working a rotating night shift at least three nights per month for 15 or more years increases the risk of CRC in women [14]. Night shift work also increases the risk of breast cancer [12] and endometrial cancer [16] in women. Therefore, researchers have considered a possible link between molecular clock machinery and some aspects of carcinogenesis such as angiogenesis, cell proliferation, apoptosis and DNA repair [23]. Indeed, aberrant expression of circadian clock genes has been observed in CRC [24], breast cancer [25], and endometrial cancer [26]; however, associations between the expression of circadian clock genes and GC have not been reported in the literature.

In this study, we observed an up-regulation of *PER2* in GCs. *PER2* play an important role in tumor suppression and DNA damage response *in vivo*[27]. Our previous studies have revealed down-regulation of *PER2* in HCC [18], CML [19,20], HNSCC [21], and breast cancer [25] but not in endometrial cancer [26]. Recently, reduced *PER2* expression has also been reported in pancreatic cancer [28] and CRC [29]. Down-regulated expression of *PER2* has been found in many cancers in both humans and mice [30,31] and often considered a tumor suppressor gene; however, we cannot explain why upregulated expression of *PER2* has, to date, been found only in GC. Indeed, the roles of circadian clock genes in the mechanism of carcinogenesis remain to be clarified. The role of *PER2* as a tumor suppressor may not be applicable in all cancers.

In this study, we also observed an up-regulation of *CRY1* in more advanced stage GC but not in earlier stage. *CRY1* is a component of the negative circadian feedback loop and is essential for the maintenance of circadian rhythm [32]. *CRY1* participates in cell cycle regulation and the cellular response to DNA damage by controlling the expression of certain cell cycle genes [33]. Deregulated *CRY1* expression has also been observed in CML [19,20] and HNSCC [21] but not in HCC [18] or endometrial cancers [26]. A 2013 study by Yu et al. found up-regulated expression of *CRY1* in CRC cancer tissues compared with that in adjacent noncancerous tissues in 168 CRC patients [34]. Higher *CRY1* expression was found in patients with lymph node metastasis and more advanced stages. The authors also found higher expression of *CRY1* correlated positively with poor patient outcomes. In vitro study, they found overexpressed *CRY1* of CRC cells promote cell proliferation and migration. In mouse study, nude mice had more obvious tumor growth after subcutaneously injecting overexpressed *CRY1* of human CRC cells compared to that in control group. Their results suggested *CRY1* plays an important role in CRC development and progression both in humans and mice, and may be a prognostic biomarker in CRC [34]. Similar to these findings in CRC, our study showed *CRY1* overexpression in more advanced GC. A statistical significance was not reached for higher *CRY1* expression indicating a poor prognosis, but the results may be limited by the small number of patients in our study. It is necessary to collect more cases in the future to validate the relationship of *CRY1* expression and GC cancer stage. *CRY1* expression may be considered a useful biomarker for determining cancer stage and prognosis in GC patients.

A correction between patients’ survival days and the expression level of *PER3* was also observed in our study. *PER1*, *PER2* and *PER3* genes belong to the same *Period* gene family. *PER1* and *PER2* are important in regulating the circadian clock [7,9,27] but the exact role of *PER3* has not been well described. It has been shown that the *PER1*, *PER2*, *PER3* and *Dec1* genes are expressed in a similar circadian manner in human peripheral blood mononuclear cells, with the peak level occurring during the habitual time of activity [35] suggesting that the oscillation of *PER3* may also be an essential factor in maintaining circadian rhythm. Besides, altered *PER3* expression has been reported in various cancers, including CML [19,20], HNSCC [21], HCC [18], and CRC [36]. Further investigations of *PER3* function may reveal the direct links between deregulation of *PER3* and prognosis in GC patients.

Down-regulation of one or more circadian clock genes has been found in most cancers, which is in contrast to our findings. Although an aberrant circadian rhythm in malignant tissues is commonly observed, what is the exact mechanism through disrupted circadian rhythm to carcinogenesis remains to be clarified. Gating of the cell division cycle by the circadian clock has been observed in some organisms [37,38] and humans. A study by Bjarnason et al. [39] found correlation with the timing of circadian clock gene expression in oral mucosa and the timing of S phase of the cell cycle, suggesting that the circadian clock may control the timing of cell-cycle events in tissues. Alteration in the circadian clock genes expression, regardless up- or down-regulation, breaks the balance of cell division and results in proliferation of tumor cells. Disrupted circadian rhythm may therefore be is both a cause and an effect of cancer.

GC is a multistep and multifactorial disease. *Helicobacter pylori* (Hp) infection is the most important factor in the pathogenesis of chronic gastritis and is an essential factor in GC. Hp-related chronic gastritis often results in atrophic gastritis and intestinal metaplasia which are indicators of an increased risk of malignant transformation and serve as precancerous markers [40,41]. Gastrointestinal disorders, mainly pain and alterations in bowel habits, are more common in shift workers than in day workers. Ulcers have been named the occupational disease of shift workers. Up to date, the association between circadian rhythm disruption and Hp-related gastritis, peptic ulcers or GC has not been well described. A recent study reported a weak correlation between shift work and Hp-positive gastritis or upper gastrointestinal complaints [42] but the results did not support the conclusion that shift work is related to gastric disorders. Studies in nocturnal animals have demonstrated that limiting food availability completely inverts the phase of the expression of circadian clock genes in peripheral tissues [43]. During caloric restriction, both the suprachiasmatic nucleus (SCN) and peripheral oscillators exhibit resetting of circadian rhythms [44]. Because circadian rhythms are directly dictated by food availability, we hypothesize that circadian rhythm disruption partly involved in the development of GC. Therefore, we first examined the expression of circadian clock genes in GC and in hopes of finding a link. Future studies analyzing the expression of circadian clock genes in Hp-positive and Hp-negative GCs would be interesting to investigate the role of Hp in gastric circadian rhythm disturbance.

Whether animal or human, studies have disrupted circadian rhythms and deregulated expressions of circadian clock genes in the cancer development and progression. We hope that the roles of circadian clock genes in the mechanism of carcinogenesis will be well clarified in the future.

## Conclusions

In this study, we observed an up-regulation of *PER2* in GC, an up-regulation of *CRY1* in cancers of more advanced stage, and a correlation between patients’ survival days and the expression level of *PER3*. Our results suggest that deregulated expression of circadian clock genes exits in GC and that circadian rhythm disturbance may be associated with the development of GC.

## Abbreviations

CML: Chronic myeloid leukemia; GAPDH: Glyceraldehyde-3-phosphate dehydrogenase; GC: Gastric cancer; HCC: Hepatocellular carcinoma; HNSCC: Head and neck squamous cell carcinoma; Hp: *H. pylori*; IHC: Immunohistochemistry; qRT-PCR: Real-time quantitative reverse transcriptase-polymerase chain reaction.

## Competing interests

The authors declare that they have no competing interests.

## Authors’ contributions

MLH, SHL, and MYY designed the study and wrote the manuscript. PML and HYHL performed the experiments. KTY collected the samples and the corresponding clinical data. CMH, YCL and HHH performed the statistical analysis. All authors have read and approved the final manuscript.

## Pre-publication history

The pre-publication history for this paper can be accessed here:

http://www.biomedcentral.com/1471-230X/14/67/prepub
